# Oligosaccharides: Defense Inducers, Their Recognition in Plants, Commercial Uses and Perspectives

**DOI:** 10.3390/molecules25245972

**Published:** 2020-12-16

**Authors:** Nathalie Guarnizo, Diego Oliveros, Walter Murillo-Arango, María Bianney Bermúdez-Cardona

**Affiliations:** 1 Grupo de Investigación en Productos Naturales de la Universidad del Tolima, GIPRONUT, Departamento de Química, Facultad de Ciencias, Universidad del Tolima, Ibagué 730006, Colombia; dfoliverosg@ut.edu.co (D.O.); wmurillo@ut.edu.co (W.M.-A.); 2 Departamento de Química, ETSEA, Universidad de Lleida, 25198 Lleida, Spain; 3 Grupo Interdisciplinario de Investigación en Fruticultura Tropical, Facultad de Ingeniería Agronómica, Universidad del Tolima, Ibagué 730006, Colombia; mbermudez@ut.edu.co

**Keywords:** oligosaccharides, plant defense elicitor, resistance induction

## Abstract

Plants have innate immune systems or defense mechanisms that respond to the attack of pathogenic microorganisms. Unlike mammals, they lack mobile defense cells, so defense processes depend on autonomous cellular events with a broad repertoire of recognition to detect pathogens, which compensates for the lack of an adaptive immune system. These defense mechanisms remain inactive or latent until they are activated after exposure or contact with inducing agents, or after the application of the inductor; they remain inactive only until they are affected by a pathogen or challenged by an elicitor from the same. Resistance induction represents a focus of interest, as it promotes the activation of plant defense mechanisms, reducing the use of chemical synthesis pesticides, an alternative that has even led to the generation of new commercial products with high efficiency, stability and lower environmental impact, which increase productivity by reducing not only losses but also increasing plant growth. Considering the above, the objective of this review is to address the issue of resistance induction with a focus on the potential of the use of oligosaccharides in agriculture, how they are recognized by plants, how they can be used for commercial products and perspectives.

## 1. Introduction

The phenomenon of resistance induction as a strategy that seeks to improve the natural defenses of plants was described for the first time in 1901 in the work of Beauverie, “Essais d’immunisation des vegetaux contre les maladies cryptogamiques” [1], followed by several studies in the early 1900s, as mentioned by Alexandersson, et al. [2], and many more recent studies, showing that it is an alternative strategy of interest [3,4,5,6,7,8,9]. However, in agriculture, its use is not common, the traditional, is genetic improvement where plant varieties have been domesticated based on fruit quality and yield, but this has resulted in reduced resistance to pathogens compared with wild varieties, so most crops are susceptible to numerous diseases [10]. In this sense, diseases continue to be responsible for considerable losses in crop production around the world [11], which is traditionally controlled by the use of chemical pesticides that today have been rejected by society for the damage it generates in crops, the environment and even to the health of farmers and consumers, thus generating a need for agriculture that seeks sustainable and environmentally friendly practices [10,12]. Therefore, a regulation has been generated in Europe, the (EC) 1107/209, complemented by directive 2009/128/EC, which forces members of the state to generate policies that contemplate plans based on an integrated management of the disease with less dependence on chemicals.

As a result of the aforementioned, resistance induction represents a focus of interest, as it is an alternative that promotes the activation of plant defense mechanisms, reducing the use of chemical synthesis pesticides, [13], an alternative that has even led to the generation of new commercial products with high efficiency, stability and lower environmental impact [14] that increases productivity by reducing not only losses but also by increasing plant growth [15].

Considering the above, the objective of this review is to address the issue of resistance induction with a focus on the potential of the use of oligosaccharides in agriculture, how they are recognized by plants, how they can be used for commercial products and perspectives.

## 2. Plant Defense Responses and Resistance Induction

Plants have an innate immune system or defense mechanisms that respond to the attack of pathogenic microorganisms [16,17]. Unlike mammals, they lack mobile defense cells, so defense processes depend on autonomous cellular events with a broad repertoire of recognition to detect pathogens that compensates for the lack of an adaptive immune system [18]. These defense mechanisms remain inactive or latent until they are activated after exposure or contact with inducing agents [19], or after the application of the inductor. The defense mechanisms remain inactive only until they are affected by a pathogen or challenged by an elicitor from the same, in which case a response has been observed that is faster or stronger with respect to the plants that did not have contact with the inductor [20].

Among the varied defense responses that the plant has, there are changes in the ion flow that lead to depolarization of the plasma membrane, production of reactive oxygen species (ROS), nitric oxide (NO), activation of calcium-dependent protein kinases or mitogen-activated protein kinases (CDPKs and MAPKs) [13,21,22,23] and activation of the octadecanoid pathway, one of the best known defense mechanisms, whose end product, jasmonic acid (JA), induces the expression of several genes [24]. In fact, together, these signaling events modulate the expression of transcription factors (TFs), activities that lead to massive transcriptional reprogramming related to defense, which in turn results in the activation of various genes that modulate various antioxidant enzymes [25,26] and metabolites specifically related to stress. These include, for example, pathogenesis-related (PR) proteins that include β-1,3-glucanases and chitinases, compounds with antimicrobial activity such as phytoalexins and deposition of callose and lignin for cell wall strengthening [10,27,28].

All of the aforementioned defense mechanisms are generated because the plant recognizes “nonnative” signals related to microbe-associated molecular patterns or pathogen-associated molecular patterns (MAMPs/PAMPs), which are constituents of the cell wall of bacteria, such as flagellin, of fungi, chitin or fragments of pathogens oomycetes (Phytophthora) in the case of β-(1,3)-(1,6)-glucan. Many MAMPs have been described and can be (glyco) type proteins, carbohydrates or lipids. In addition to the signals that come from microorganisms, there are also those associated with the plant, from the host itself, the damage-associated molecular patterns (DAMPs), which are very structurally diverse and include, for example, pectin-derived oligogalacturonides. Once MAMPs/PAMPs are recognized by pattern-recognition receptors (PRRs), located in the plasma membrane or in the cytoplasm, a wide variety of responses related to MAMP/PAMP-triggered immunity (MTI/PTI) are triggered [13,18,29,30].

In addition to the innate immune system, plants can also acquire immunity against the perception of specific biotic and abiotic stimuli, a process mediated by the priming of inducible defenses, which causes plants to show an activation of various rapid or strong cellular defense responses or both [20]. In this sense, two types of induced resistance have been identified and widely studied: systemic acquired resistance (SAR) and induced systemic resistance (ISR). The first is a state of defense induced by a local infection with pathogens or by the application of a chemical substance that confers resistance to a broad spectrum of pathogens and is dependent on salicylic acid (SA) [31] Therefore, when this process occurs, greater resistance is observed in the distal parts as a consequence of mobile signals in the plant belonging to the SA, which is biologically active in tissues far from the point of origin from where the response began [32], which is related to localized necrosis, can present as a hypersensitive response or local necrotic lesion caused by a virulent pathogen [33,34]. The induction of SAR requires the accumulation of endogenous salicylic acid signals, which mediate the activation of a wide set of pathogenesis-related (PR) genes. It has also been known that SA can have a dual role in signaling: activation of PR genes or low doses of SA that do not activate defense genes directly can prepare the tissue for an enhanced expression of defense genes in a subsequent infection of the pathogen. In fact, in some cases, it has been found that there is no response to induction with SA until the plant is inoculated with the pathogen or its components [35].

Regarding induced systemic resistance (ISR), this appears when there is a beneficial interaction between plants and microorganisms, the latter helping plant nutrition but also overcoming biotic or abiotic stress in addition to improving the defense capacity of the plant and effectively preventing a broad spectrum of pathogens [36]. This type of induced resistance is considered independent of SA and is not associated with major changes in the expression of defense genes, probably because this would lead to a strong investment in resources and reduction of host fitness, as has been shown in previous research [20,37,38]. In this sense, it is widely known that ISR is governed by jasmonic acid (JA) and ethylene (ET) [34], although it may not alter the production of JA or ET, as mentioned by Pieterse, et al. [39], in which case the effect could be more related to improved sensitivity to these plant hormones.

Thus far, the defense mechanisms of plants and the types of resistance induction have been mentioned, but what inducers are has not been conceptualized. Plant resistance inducers (PRIs) of exogenous application are interesting because they can be incorporated into integrated disease management programs. These inducers are also called elicitors, although sometimes the terms are confused. Elicitors, in a broad sense, are chemicals or biofactors from various sources that can induce physiological and morphological changes in the target organism. These include abiotic elicitors, such as metal ions and inorganic compounds, and biotic elicitors from fungi, bacteria, viruses (or fragments of these), components of the plant cell wall as well as chemicals that are released at the site of attack by plants after the emergence of pathogens or herbivores [40]. In the same way, other authors define that an elicitor or inducer is a molecule, or molecules, present in an organism or produced by the plant itself, whose function includes the generation of defense responses [19]. β-1,3-glucan of *Septoria tritici* triggered wheat defense responses related to the production of β-1,3-glucanases and callose deposition to strengthen the cell wall [41] or the effect that the extract from *Phaseolus vulgaris* leaves had on the same plant, generating a strong defense response related to the production of reactive oxygen species [42]. However, it is important to note that in the field of crop protection, they offer many advantages, because they do not attack the pathogen as such (they are not directly toxic to pathogens, which is the basis of pesticides, so they do not generate resistance); however, they are recognized by the receptors of the plant membrane and induce innate immunity [13]. They can potentially protect against multiple pathogens [43], mainly because they do not have a specific mechanism of action [14].

In general, it should be noted that the results of resistance induction will depend on the genotypes of both pathogens and hosts, which results in variable responses where the relationship between the level of resistance and the level of resistance induction is not clear, although in most cases, it has a direct relationship [44,45]. Likewise, an important issue regarding the induction of resistance is the influence of the environment and genotype on the plant’s response capacity to defense inducers, which was shown by Bruce [46], where the attack of herbivores could promote the phenotypes of defense induction through generations and epigenetic change may be the basis of its lasting effect.

## 3. Oligosaccharides as Defense Inducers

Some molecules that are used as elicitors are therefore PAMPs/MAMPs that, as mentioned above, can be of different types, such as oligosaccharides (OGAs), on which research has focused in recent times, given that a large amount of these mediate the pathogenesis [47]. In this context, the knowledge of different glucans that are part of fungi, oomycetes or bacteria presents a new perspective to not only analyze and understand plant-pathogen interactions but also to determine the potential of these as defense-inducing agents for the protection of plants.

In this sense, it has been shown that some oligosaccharides can act as elicitors and consequently activate the defense responses of plants. This has been studied in different plants, and for this review, studies that have used oligosaccharides, as well as the use of some chemical elicitors, are referenced (Table 1) in order to show evidence that the responses shown by the plants with any of the elicitors are similar; therefore, it is possible to consider the oligosaccharides as elicitors that can be used in agriculture.

**Figure 1:**
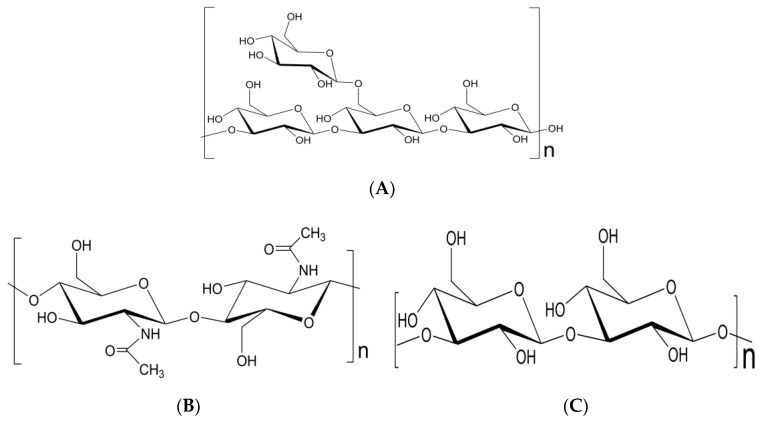
Oligosaccharides used in resistance induction: (**A**) Laminarin, (**B**) Chitin, (**C**) Curdlan.

Regarding the origin, the oligosaccharides involved in plant-pathogen interactions are produced by enzymatic degradation of polysaccharides that are structural constituents of the cell wall of fungi or plants or pathogenicity factors of pathogens [48]. The activity of these compounds is dependent on the dose but also on the degree of polymerization (DP) [49,50], without consensus in this regard. In the case of tobacco cells, laminarin (MW: 504.4 g/mol), a storage β-glucan composed of glucose, and mannitol (Figure 1A), from brown algae, which has an average of 25–33 DP and up to three β-glucose branches in position 6, induces defense responses slower than other β-1,3-glucans with low DP (2–10) [5]. Given that other studies showed contradiction with these results, as in the case of chitin (MW: 203.1925 g/mol), a linear polymer of N-acetylglucosamine (Figure 1B), with higher activity reported for DP 7–8 and low or none for DP < 5 [51], or curdlan a linear polymer of glucose (Figure 1C), which showed a marked effect with DP 5–7 with respect to those of DP3 [52]. We sought to have greater clarity in this regard andwe found that it is important to also take into account other factors, such as the type of polymer used and the pathosystem (on which the activity of one molecule or another depends highly), namely, for a lower OGA size, a better response of the plant is generated, while with chitosan, DP between 7–10 is usually more active [50].

Regarding the aforementioned pathosystem towards which the process of induction of resistance with oligosaccharides is directed, it is precisely this that seems to determine the way in which the plant responds to structurally different β-glucans, a response that must have evolved in these plants and that makes them react against one or another molecule [50]. In the case of rice, the recognized β-glucans are branched [48], while tobacco reacts to linear β-1,3-glucans [5,61], and soybeans that were initially thought to only recognize branched oligosaccharides have actually been shown to also recognize DP 3 linearly [60,65,66].

In addition, acetylations, methylations and degrees of sulphation influence the biological activity of these biomolecules. For example, the CERK1 receptor, which is a chitin receptor [29] and triggers defense responses, recognizes partially deacetylated chitin but not fully deacetylated chitin [67]. Regarding the degree of methyl esterification of oligosaccharides modulated by the enzyme pectin methyl esterase (PME), this is crucial for the activity, that is, the overexpression of the enzyme reduces the degree of methyl esterification of OGA and with it, a reduction of symptoms caused by *Botrytis cinerea* in Arabidopsis and strawberries [68,69]. For sulfate groups, when they are substituents in biomolecules, which generally implies greater biological functionality, these substituents modify the three-dimensional structure of the molecule, making it more resistant to degradation by β-1,3-endoglucanases and exoglucanases [50]. Its ability to improve defense responses has been evaluated in vine plants [49] and tobacco [61], to cite some examples.

## 4. Recognition of Polysaccharides in Plants

Protein glycosylation is generally recognized as one of the major co- and post-translational modifications. This interaction between carbohydrates and their associated protein mediates important biological events. These proteins of nonimmune origin, which bind to specific carbohydrate structures, are classified as lectins. The location of these abundant proteins in the extracellular space or vacuoles suggests a role in plant defense [70]. They are found at a basal level but are inducible; that is, they are overregulated in response to biotic or abiotic stress [71].

**Figure 2:**
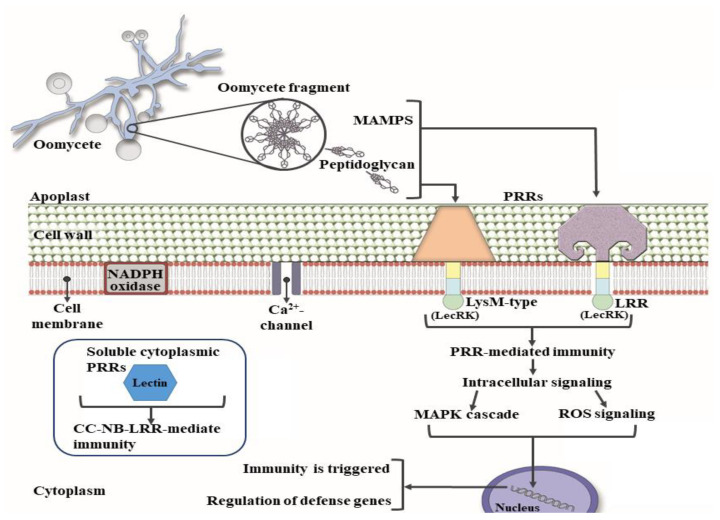
Recognition of effectors in plants by pattern-recognition receptor (PRR) lectins. Once the plant perceives the peptidoglycans (MAMPs) through PRRs with a lectin domain (LecRK and lectin-type lectin-kinase receptor), an intracellular signaling cascade is generated downstream, which leads to protein phosphorylation, generation of reactive oxygen species (ROS), regulation of phytohormones, activation of transcription factors due to what is known as microorganism-/pathogen-triggered immunity (M/PTI) or, when soluble receptors recognize effectors from the pathogen, the generation of effector-triggered immunity (ETI).

In this sense, the first inducible line of defense common to plants and animals is MAMP-triggered immunity and PAMP-triggered immunity (MTI/PTI) [16]. MTI or PTI is based on the recognition of highly conserved structures for microorganisms that are not present in the host organism. In plants, these MAMPs/PAMPs are detected on the surface by pattern recognition receptors (PRRs) and as many carbohydrate structures, such as peptidoglycans (PGN), have been established as MAMPs/PAMPs [18,72,73] Thus, lectins seem to play an important role as PRRs. These PRR lectins are highly variable in structure and may be soluble but also associated with the membrane [70] (Figure 2). It seems that they recognize long fragments of the PGN sugar skeleton. Although the specific size that this fragment must have to trigger immunity has yet to be established [73], what has been evidenced is its effect on primers of defenses, for example, in tomato plants, which acquired a greater resistance to the infection caused by *P. syringae* pv. *tomato* [74]. Regarding soluble receptors, it is important to clarify that these receptors recognize the presence of cytoplasmic effectors; these receptors are named for their conserved nucleotide-binding and leucine-rich repeats (NB-LRR) domains [75] (Figure 2).

Many of the lectin-type receptors described so far in plants belong to the lysine motif protein family (LysM) (Figure 2); among the first identified are the LysM domain protein (LYM 1) in Arabidopsis and AtLYM3, two PGN-binding proteins bound to glycosylphosphatidylinositol (GPI) and the chitin receptor (AtCERK1), which does not bind to PGN but is required for signal transduction in Arabidopsis [76,77]. In the same way, many other families have been identified, such as Hevein (*Hevea brasilensis*), Jacalin (jackfruit) and from legumes that recognize chitin, mannose/galactose and mannose, respectively [70].

PRRs translate one or more extracellular lectin domains, which are frequently coupled to an intracellular Ser/Thr kinase domain. Therefore, lectin receptor kinases (LecRK) can be classified into four types: G-, C-, L- and LysM. The G-type LecRKs have a lectin domain that resembles agglutinin, the C-type LecRKs are calcium-dependent, the L-type LecRKs (legumes) are the most numerous group of kinase receptors, and LysM is the most studied. One of its domains contains a lysine that binds to several bacterial peptidoglycans and fungal chitin, as previously mentioned [78].

Similarly, for the specific case of oomycete pathogens, there are effectors (Avr avirulence factors) that activate R genes according to the gene-by-gene model; this effector is recognized by specific receptors (R proteins), a process that leads to a hypersensitive response (HR) [16]. To delve a little further, reference will be made to research on *Phytophthora infestans*, because it is one of the most studied pathogens in reference to this recognition process. In this way, R genes typically encode immune receptor proteins, called supercoiled helix proteins of the coiled coil, nucleotide binding, leucine rich repeat (CC-NB-LRR) [79,80]; these proteins have important biological functions, such as the regulation of gene expression. In this case, as a molecular trap, it detects effectors directly or by manipulation of target proteins performed by the effector that are monitored by the receptor as such [81,82]. In contrast, all the oomycete effector proteins known so far contain an RXLR motif (Arg-Xaa-Leu-Arg) that is believed to mediate the delivery of the effector to the host cell, although the mechanism by which this occurs continues to be a matter of discussion [83]. These effectors perform various functions, mainly promoting the virulence of the pathogen by suppressing plant immunity [75,84].

## 5. Oligosaccharides as a Disease Management Strategy: Commercial Use

Alternatives to commercial fungicides have emerged over the past few years. In 2014, a group of scientists with knowledge on the possible advantages of working with oligosaccharides as resistance inducers set out to create the company FytoFend SA, which works with two main approaches. The first is the optimization of the production of elicitors, and the other is the study of their mechanisms of action. Their efforts can be seen today in various products that are successfully marketed for crop protection; one product is FytoSave^®^, composed of a complex of oligochitosans and oligopectates as an active substance, which was tested in a growing crop against downy mildew of grapes and cucumber. It was found that when it was applied by spraying in an interval between 7–14 days, it reduced the severity of the disease. Additionally, the product persisted and showed a cumulative effect. On the other hand, it could be perfectly combined with traditional chemical management without problems and with some interesting advantages, such as not having a preharvest interval (for some chemicals, such as bitertanol applied in cucumber, it can be three days and three weeks for the triadimenol), reducing application risks for the operator and the environment, increasing their market valuation and, most importantly, reducing the risk of increasing fungicide resistance [85].

In later developments, COS-OGA was tested against potato late blight caused by *Phytophthora infestans*, resulting in increased resistance to the disease due to leaf spray, which could be corroborated with the analysis of gene expression, where an increase of two pathogenesis-related proteins, PR-1 and PR-2, was observed. The first is a protein known to be linked to partial resistance to *P. infestans* in species of the genus *Solanum*, and the second encodes a β-glucosidase with the ability to hydrolyze β-1,3-glucan, the largest component of the cell wall of oomycetes but also a protein that appears to be linked to the pathway of salicylic acid (SA), a compound required in the defense of plants [3]. In fact, in a previous study, it was reported that the induction of immunity in plants due to the COS-OGA elicitor is a cumulative process involving SA. The plant had no modification of JA and ET but did show an upregulation of PR proteins, proteases and increased peroxidase activity, suggesting a mechanism of action of the systemic acquired resistance (SAR) of the elicitor [86].

On the other hand, the Spanish Lida Plant Research SL sells what they have called “phytovaccines,” among which is FytoSave^®^ and Stemicol^®^. About the first product, some things were mentioned above, while about the second one, it should be noted that it is a chito-oligosaccharide that has an effect on the reduction of fruits with rot, in the case of tomato, strawberries and grapes, and benefits such as increased production of paprika, onion and potato [87]. In general, Lida defines phytovaccines as “substances capable of optimizing the expression potential of genes related to defense to the maximum,” and they have a blog where they record different reports from many farmers who have had good results in the control of powdery mildew in zucchini and pepper and mildew in table grapes. Recently, an alliance between Lida Plant Research and FytoFend SA managed to make the FytoSave^®^ product the first plant phytovaccine with phytosanitary registration admitted by the European Commission for use in organic agriculture in 2018, and its active component COS-OGA, is listed by the same commission as low risk [88], all of which allows us to see that the interest in oligosaccharides for resistance induction is a topic of interest that has a promising future, since the current world is moving towards ecological agriculture that uses fewer chemicals in its production.

## 6. Perspectives

The induction of resistance is, therefore, an alternative that seeks to restrict or eliminate chemically synthesized pesticides, which currently requires a considerable investment per hectare with respect to the total production cost of each crop. In addition, it is clear that the trend of consumers today is inclined towards foods that are produced in a way that is environmentally sustainable, using fewer pesticides in a way that balances the economic, environmental and quality of life benefits not only for farmers but also for consumers [89]. This type of agriculture is, therefore, a differential factor in the market that is well valued in economic terms, so that a product that in its chain has required a lower use of pesticides will have a higher price, which ultimately translates into a more competitive producer, with a product with less traceability and better acceptance on international markets.

On the other hand, as mentioned, plants have various ways of recognizing a pathogen; namely, structural changes caused by a pathogen or effectors of the pathogen that disturb or modify proteins of the plant. This information has become relevant and could be a very useful tool in crop protection. For the construction of new molecules or assays with B-glucans or other oligosaccharides from different sources, a plant, when recognized by its defense mechanism, improves its defense responses. In turn, this increases the possibility to isolate and transfer receptors between species, paths that have a great potential to generate durable resistance, showing good results.

## Figures and Tables

**Table 1:** Inductors of chemical synthesis and oligosaccharides that generate resistance in different cultures against their respective pathogens.

Inductor	Plant	Disease	Pathogen	Application	Response	Quote
**Chemical synthesis**						
Acibenzolar-S-methyl (ASM)	Cape gooseberry	Vascular wilt	*Fusarium oxysporum*	Foliar	Greater accumulation of defense proteins and signal molecules in the root.	[53]
Acibenzolar-S-methyl (ASM)	Jimson weed *Nicotiana benthamiana*	Yellow spot	Iris yellow spot virus (IYSV)	Sprayed	Expression of PR proteins, fewer lesions and reduction in virus levels quantified by PCR.	[54]
Β-aminobutyric acid (BABA)	Tomato	Gray mold disease	*Botrytis cinerea*	Application to the soil of the compound	Upregulation of defense-associated compounds such as phenylpropanoids.	[9]
Hexanoic acid	Orange	Canker	*Xanthomonas citri* subsp. *citri*	Sprayed and soaked soil	Reduction of lesions, improved expression of PR proteins and callus deposition.	[55]
Salicylic acid	Green soybeans	Virosis	Mungbean yellow mosaic India virus (MYMIV)	Sprayed	Stimulation of SOD and GPX enzymes. Activation of defense-related proteins, increased phenolic and H_2_O_2_ content.	[6]
Salicylic acid	Rice	Blight of rice	*Magnaporthe oryzae*	Sprayed	Activation of proteins involved in functions such as defense, antioxidant enzymes and signal transduction. Increase in the levels of reactive oxygen species.	[7]
Salicylic acid	Avocado	Root rot	*Phytophthora cinnamomi*	Submerged root	Production of the phenol-2,4-bis (1,1-dimethylethyl) compound, with antifungal activity and against *P. cinnamomi*.	[56]
Salicylic acid (SA) and Methyl jasmonate (MeJA)	Tomato	Wilt	*Fusarium oxysporum* f. sp. *lycopersici*	Daily spray for three days	Increased activity of antioxidant enzymes, decreased lipid peroxidation.	[8]
Riboflavin	Rice	Rice pod blight	*Rhizoctonia solani*	Sprayed	Priming of the expression of lipoxygenase, key in the octadecanoid pathway, upregulation of the PAL enzyme, and therefore, an improved lignification.	[57]
Silicon (Yes)	Paprika		*Phytophthora capsici*	Soil	It reduced the severity and improved the development of the plant.	[58]
Copper sulfate pentahydrate (Phy)	Cotton		*Fusarium oxysporum* sp. *Vasinfectum*	Foliar and seeds	Low severity index.	[59]
**Oligosaccharides**						
Oligosaccharides isolated from *Ulva lactuca*	Tomato	Wilt	*Fusarium oxysporum* f. sp. *lycopersici*	Internodal injection in the middle part of seedlings	Stimulation of PAL activity accompanied by increased phenolic compounds and the induction of salicylic acid.	[4]
Curdlan oligosaccharide (CurdO)	Potato	Late blight	*Phytophthora infestans*	Infiltration	Expression of defense-related proteins, reduction of leaf lesions, higher concentrations of salicylic acid, higher PAL, GLU and CTN activity and better yields.	[52]
Oligogalacturonides	Arabidopsis		*Pectobacterium carotovorum* sp. *Carotovorum* SCC1	Foliar	Activation of defense-related genes among which are groups associated with phytohormones, oxylipin biosynthesis, programmed cell death and other signaling.	[60]
Laminarin (β-1,3-glucan)	Tobacco	Bacteriosis	*Erwinia carotovora* subsp. *carotovora*	Addition of elicitor to cell culture	Accumulation of PR proteins, release of H_2_O_2_, stimulation of PAL and LOX activity and accumulation of SA.	[61]
Chito-oligosaccharides (COS)	Blackberry	Biotic and abiotic stress		Foliar spray	Higher phenolic content and greater free radical trapping capacity (ABTS), as well as increased succinate dehydrogenase activity.	[62]
Curdlan-oligosaccharides (β-1,3-glucan (CRDO)) and Laminarin	Tobacco	Tobacco mosaic	Tobacco mosaic virus (TMV)	Addition of elicitor to cell culture	Production of nitric oxide, greater movement of stomata with CRDO and greater protection against the virus in the case of laminarin.	[5]
Tramesan	Durum wheat	Nodorum blotch	*Parastagonospora nodorum*	Sprayed	Pro antioxidant molecule, induced the expression of oxidative stress defense-related genes.	[63]
Tramesan	Durum wheat	Septoria Leaf Blotch complex (SLBC)	*Parastagonospora nodorum* *Zymoseptoria tritici*	Sprayed	Increased the levels of jasmonic acid and the early expression of plant defense genes.	[64]
